# Synthesis and Characterization of Doped Magnesium Hydroxide for Medium Heat Storage Application

**DOI:** 10.3390/ma16186296

**Published:** 2023-09-20

**Authors:** Nawaf Albeladi, Anti Kur, Robert Mokaya, Jo Darkwa, Sarah Roger-Lund, Mark Worall, John Calautit, Rabah Boukhanouf

**Affiliations:** 1 School of Chemistry, University of Nottingham, Nottingham NG7 2RD, UK; robert.mokaya@nottingham.ac.uk; 2 Department of Chemistry, Taibah University, Yanbu Al Bahr 46423, Saudi Arabia; 3 Department of Architecture and Built Environment, University of Nottingham, Nottingham NG7 2RD, UK; jo.darkwa@nottingham.ac.uk (J.D.); enysr5@exmail.nottingham.ac.uk (S.R.-L.); lazmw@nottingham.ac.uk (M.W.); ezzjkc@exmail.nottingham.ac.uk (J.C.); lazrb@exmail.nottingham.ac.uk (R.B.)

**Keywords:** thermochemical energy storage, magnesium hydroxide, composite material, characterization, dehydration temperature

## Abstract

The amount of waste heat generated annually in the UK exceeds the total annual electricity demand. Hence, it is crucial to effectively harness all available sources of waste heat based on their varying temperatures. Through suitable technologies, a substantial portion of this waste heat has the potential to be recovered for reutilization. Thermochemical energy storage (TCES) provides the best opportunities to recover waste heat at various temperatures for long-term storage and application. The potential of TCES with magnesium hydroxide, Mg(OH)_2_, has been established, but it has a relatively high dehydration temperature, thus limiting its potential for medium-temperature heat storage applications, which account for a vast proportion of industrial waste heat. To this end, samples of doped Mg(OH)_2_ with varying proportions (5, 10, 15, and 20 wt%) of potassium nitrate (KNO_3_) have been developed and characterized for evaluation. The results showed that the Mg(OH)_2_ sample with 5 wt% KNO_3_ achieved the best outcome and was able to lower the dehydration temperature of the pure Mg(OH)_2_ from about 317 °C to 293 °C with an increase in the energy storage capacity from 1246 J/g to 1317 J/g. It also showed a monodisperse surface topology and thermal stability in the non-isothermal test conducted on the sample and therefore appears to have the potential for medium heat storage applications ranging from 293 °C to 400 °C.

## 1. Introduction

Concerns about carbon dioxide (CO_2_) emissions and global warming are lingering issues that have attracted a lot of attention across nations. According to published climate data, over 34 billion tonnes of CO_2_ are currently being emitted, compared to 22 billion in 1990 [1]. One of the main factors contributing to this CO_2_ output is the energy consumption in buildings, which is responsible for 40% of global energy consumption. For instance, UK buildings account for about 35% of final energy consumption and CO_2_ emissions [2]. A majority of this energy consumption is for hot water and space heating purposes [3]. Concurrently, a significant quantity of excess thermal energy is produced each year by industrial and power sources, reaching an estimated 391 TWh. This amount surpasses the UK’s entire annual electricity consumption by 35% [4]. Several research efforts have targeted cutting down the country’s carbon footprint as much as possible, and one promising way to do this is by improving the efficiency of energy usage. A key step in this direction is to effectively recover waste heat for reuse. Heat (or thermal) storage is identified as a crucial method for achieving this and has drawn a lot of interest and promise.

Waste heat can be categorized based on its temperature: low grade (room temperature to 250 °C), medium grade (250 °C to 500 °C), and high grade (above 500 °C). By implementing suitable energy storage methods, a significant amount of this surplus energy has the potential to be reclaimed for useful applications in the building sector. Essentially, heat is stored either as sensible heat in storage media, latent heat in phase-change materials, or in thermochemical storage due to the rearrangement of chemical bonds. However, the two major advantages of thermochemical energy storage (TCES) over the other techniques lie in their high energy storage density and zero heat losses during storage, which can enable the design of compact storage units and long-term storage possibilities [5]. Through the TCES approach, heat storage and release are conducted through reversible chemical reactions, and the heat storage amount is determined by the enthalpy change of the reaction. Thermochemical energy storage (TCES) in inorganic hydroxides has attracted growing research interest due to their relatively higher energy storage density, reversibility, and potential for efficient heat management in various applications such as renewable energy systems [6]. However, most past studies focussed more on exploring either the low-temperature (below 200 °C) or high-temperature (above 500 °C) storage materials. For instance, the low-temperature studies focussed on applications requiring heat within a temperature range of 30–150 °C. At the material level, the emphasis was on solid–gas reactions with water as sorbate in application to salt hydrates and adsorbents such as zeolites, aluminophosphates, and metal–organic frameworks [7,8], whereas hydrides, carbonates, ammonia, organic, and redox systems were applied to high-temperature (500–1000 °C) reversible reactions, especially in solar thermal processes for concentrated solar plants [9,10], thereby creating a gap in materials for medium-temperature-range applications. Out of the total projected waste heat potential of 300 TWh/year in Europe, more than a quarter exists within the 200–500 °C (medium-grade heat) range [11].

The reversible TCES reactions involving a magnesium hydroxide and magnesium oxide pair, Mg(OH)_2_/MgO, are identified as very promising for such medium-temperature applications, and many research efforts have been dedicated to investigating chemical heat storage using Mg(OH)_2_. However, earlier works by Kato et al. [12,13] indicated that its dehydration temperature was above 350 °C, which is not thermodynamically rewarding for application. Consequently, a need to lower the dehydration temperature to cover a wider temperature range arose, and an investigation by Ryu et al. [14] revealed that it should be possible to substantially alter the dehydration temperature by doping Mg(OH)_2_ with alkali metal salts. Shkatulov et al. [15] later showed that nitrate salts could have effective dehydration outcomes as dopants in hydroxides. They suggested that the nitrate anions could infiltrate the hydroxide brucite lattice in a manner that aids the formation of MgO nuclei, hence causing the structure to decompose at a lower temperature [16].

Shkatulov et al. [16] therefore doped Mg(OH)_2_ with sodium nitrate (NaNO_3_) and achieved a reduction in the dehydration temperature of up to 50 °C, albeit with heat storage decreasing from 1325 J/g to 1040 J/g. In another investigation, Shkatulov and Aristov [17] doped Mg(OH)_2_ with lithium nitrate (LiNO_3_) and obtained a reduction in the dehydration temperature of 76 °C. Sun et al. [18] obtained a reduction of 29 °C with cerium nitrate (Ce(NO_3_)_3_) as a dopant but the heat storage capacity was compromised. Li et al. [19] achieved a reduction in the dehydration temperature of Mg(OH)_2_ of 56 °C by incorporating 10 wt% of LiNO_3_. In all these investigations, the respective heat storage capacities were very much affected. Therefore, we are motivated by the need to further explore another type of dopant that could protect the energy storage integrity of Mg(OH)_2_, that is, reducing its dehydration temperature without trading off the energy stored. This marks the novel approach in our work. Potassium nitrate (KNO_3_) appears to be an appropriate dopant since it possesses higher ionic mobility as compared to other alkali metal ions [20]. The higher mobility level could therefore facilitate the diffusion of protons (H^+^) and hydroxide ions (OH^−^) during the dehydration process, thereby contributing to the acceleration and lowering of the dehydration temperature. However, to the best of our knowledge, KNO_3_ has not been previously tested as a doping agent in Mg(OH)_2_. Therefore, the primary aim of this investigation is to synthesize and characterize the composite Mg(OH)_2_/KNO_3_, envisioned as a promising thermochemical energy storage material for waste heat recovery.

This paper presents the material synthesis procedure and characterization techniques in Section 2. The results are presented and discussed in Section 3, where Section 3.1 assesses the doping process. Section 3.2 and Section 3.3 assess the thermal performance of the materials, from which the best material undergoes further physical tests in Section 3.4, Section 3.5, Section 3.6 and Section 3.7. A succinct conclusion is then presented in Section 4.

## 2. Materials and Methods

### 2.1. Material Development

**Figure 1:**
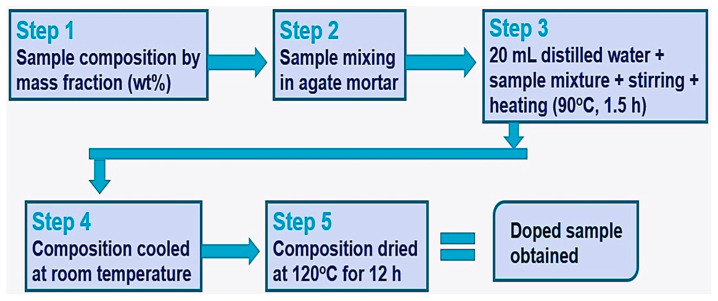
Experimental flow diagram for the doping procedure.

The composite materials were prepared by doping Mg(OH)_2_ of 95.0 % purity with KNO_3_ of 99.0 % purity (all purchased from Sigma-Aldrich, Gillingham, UK) in various mass ratios. Four different mixtures of Mg(OH)_2_ and KNO_3_ composites were prepared, containing 5%, 10%, 15%, and 20% KNO_3_ relative to the total mass, which was fixed at 10 g for all mixtures. The amounts of KNO_3_ in each composite were 0.5 g, 1 g, 1.5 g, and 2 g for the 5%, 10%, 15%, and 20% composites, respectively. Initially, the KNO_3_ and Mg(OH)_2_ powders were thoroughly mixed using an agate mortar for approximately 10 min to ensure a uniform blend. Subsequently, the mixture was transferred to a beaker for liquid mixing with 20 mL of distilled water. This was then stirred continuously at moderate speed to ensure steady mixing while being heated at 90 °C for 1.5 h. Finally, the composition was allowed to cool at room temperature before drying in the oven at 120 °C for 12 h to obtain the synthesized composite. Figure 1 shows the flow diagram for steps in the doping process.

For ease of reference, the labels MH. MH-PN5, MH-PN10, MH-PN15, and MH-PN20 are used to represent the pure Mg(OH)_2_, 5 wt%, 10 wt%, 15 wt%, and 20 wt% KNO_3_-doped Mg(OH)_2_ samples, respectively.

### 2.2. Material Characterization

#### 2.2.1. Powder X-ray Diffraction

Powder X-ray diffraction (XRD) testing provides information on structures, phases, crystal orientations (texture), and other structural parameters within materials. It was therefore carried out for phase identification of various elements within the composite samples. The test was conducted at room temperature using a PANalytical X’Pert PRO diffractometer (Malvern Instruments Ltd., Worcestershire, UK) with CuKa radiation (l = 1.5406 Å, 40 kV, 40 mA) for values of 2-Theta,0.02 step size, and 50 s step time in the range 2°–70°.

#### 2.2.2. Differential Scanning Calorimetry

Differential scanning calorimetry (DSC) measurements were taken to estimate the heat storage and dehydration temperatures for the samples. By using a TA Thermal Instrument (TA Instruments, New Castle, DE, USA), a baseline measurement was taken in the range of 25 °C to 550 °C at a heating rate of 10 °C/min on each sample weighing 5 mg. Nitrogen was used as the purge gas at a volumetric rate of 20 mL/min under 1 atm pressure.

#### 2.2.3. Thermogravimetry Analysis

Thermogravimetric analysis (TGA) was performed on each sample weighing 20 mg with a vacuum-tight thermo-microbalance SDT600 (TA Instruments, New Castle, DE, USA) to determine their thermal characteristics. This was achieved by measuring the amount of weight change in the samples as a function of increasing temperatures under nitrogen gas protection, covering a heating range of 25 °C to 600 °C at a heating rate of 10 °C/min.

#### 2.2.4. Brunauer–Emmett–Teller Analysis

Brunauer–Emmett–Teller (BET) analysis was conducted to measure the surface areas of the samples and to assess how they would interact with their environment. These were obtained via nitrogen sorption isotherm by using a Micromeritics 3Flex analyzer (Micromeritics Instrument Corporation, Norcross, GA, USA) at −196 °C. Prior to the analysis, the samples were degassed at 100 °C for 16 h under a vacuum. The specific surface areas were calculated using the BET method from the nitrogen adsorption data within the relative pressure (P/P_0_) range of 0.05–0.30. The total pore volume was estimated using total nitrogen adsorbed at relative pressure close to saturation (P/P_0_~0.99). The pore size distribution (PSD) was determined using non-local density functional theory (NL-DFT) applied to nitrogen isotherm data.

#### 2.2.5. Scanning Electron Microscopy

Surface images of the samples were taken to observe their structure. This was achieved by means of a JEOL 6490LV (JEOL Ltd, Tokyo, Japan) scanning electron microscope (SEM), operated in high-vacuum mode at 15 kV, and with a secondary electron detector. The electron beam in the vacuum excites electrons from the surface to create different signals of the material’s topology.

#### 2.2.6. Energy-Dispersive X-ray Spectroscopy

Energy-dispersive X-ray spectroscopy (EDX) is an extended functionality of the JEOL 6490LV (SEM) instrument used to obtain information on the chemical composition of samples. To do this, the material was irradiated with a high-energy beam to stimulate X-ray emission. The characteristic of the emitted radiation was then compared to the atomic energy of the matched element. During the measurement, two different areas were sampled to assess the atomic weight distribution, and the corresponding peaks were obtained.

## 3. Results and Discussion

### 3.1. Powder XRD

**Figure 2:**
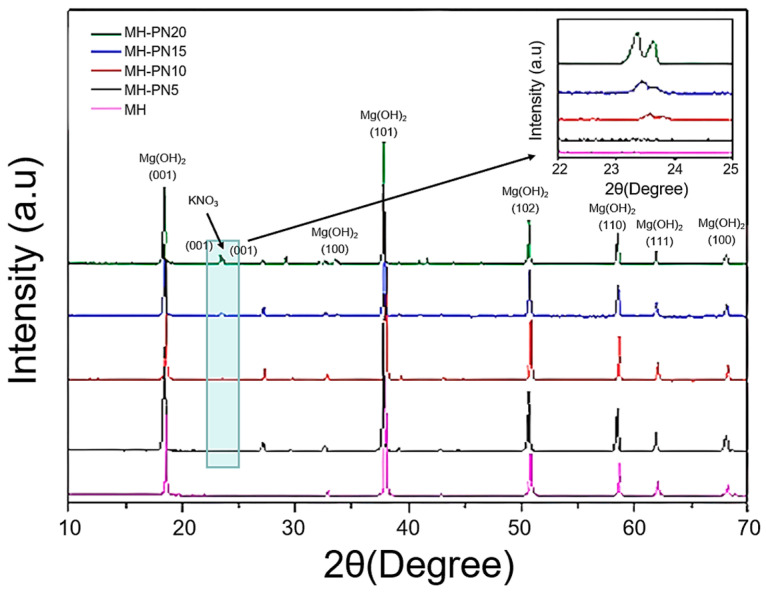
Powder XRD patterns for pure and doped Mg(OH)_2_.

The synthesized materials were characterized for phase identification of the composite elements. The XRD diffractograms for the pure and doped Mg(OH)_2_ samples are shown in Figure 2. It can be seen that pure Mg(OH)_2_ showed only a single phase (peak), whereas the doped composites showed two distinct peaks of Mg(OH)_2_ and KNO_3_. The planes agree with the standard Powder Diffraction Form (PDF) available in the Inorganic Crystal Structure Database (ICSD), references 01-074-2220 for pure Mg(OH)_2_ brucite and 00-001-0493 (standard KNO_3_). The highlighted areas show the peak positions of the KNO_3_ and the inset in the figure shows the increasing intensity of the peaks with an increase in the dopants’ proportion. The peak intensities are determined by the distribution of atoms within the material, thus enabling phase identification for the samples. Since the phases of both Mg(OH)_2_ and KNO_3_ were clearly detected as intended, this confirms that the doping process was successful.

### 3.2. DSC

**Figure 3:**
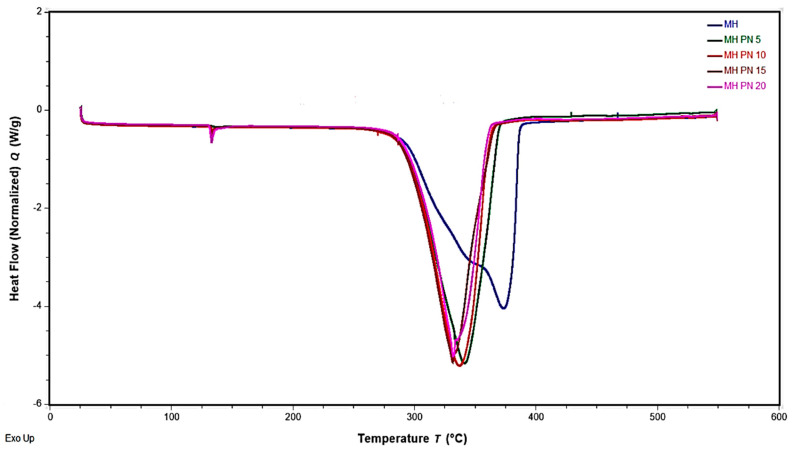
DSC overlay curves of pure and doped Mg(OH)_2_ samples.

**Figure 4:**
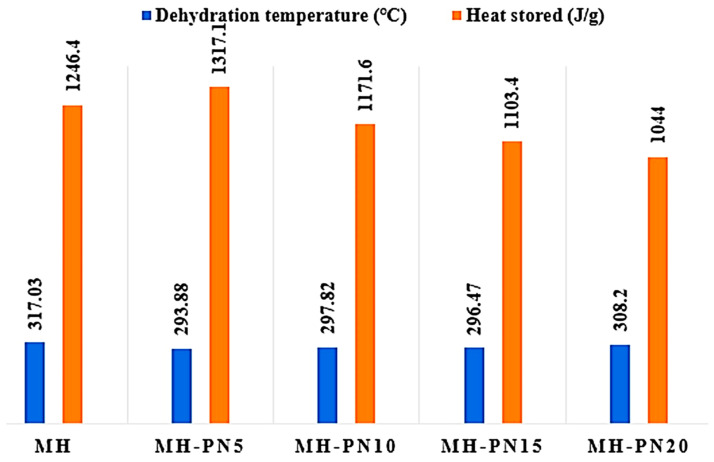
The dehydration temperatures and heat stored in the pure and doped Mg(OH)_2_.

Figure 3 displays the DSC overlay curves representing both pure and doped Mg(OH)_2_ samples. The outcomes are summarized in the bar chart (Figure 4), presenting the corresponding energy storage densities and the temperatures at which dehydration commenced. Evidently, the MH-PN5 sample, containing 5 wt% KNO_3_, exhibited the lowest onset temperature for dehydration, measured at 293.82 °C. This value is notably 23 °C lower than the dehydration onset temperature of pure Mg(OH)_2_. Impressively, MH-PN5 also registered the highest energy content of 1317.90 J/g, which is roughly 6% greater than that of pure Mg(OH)_2_. The addition of KNO_3_ could have a catalytic effect on the dehydration of Mg(OH)_2_. In this case, the presence of a small amount (5 wt%) of KNO_3_ could enhance the dehydration reaction of Mg(OH)_2_, leading to a more efficient removal of water molecules. This increased reaction rate could have resulted in higher heat absorption during the process, manifesting as a higher endothermic heat signal in the calorimetric measurement. This indicates that a small amount of salt is enough to produce a significant modifying effect. Small peaks were noticed around 132 °C and progressed with increasing dopant proportion beyond 10 wt%, which could be attributed to the excess unreacted nitrate salt in the matrix of the samples [15]. A comparable occurrence is documented in the existing literature and was ascribed to the fusion of crystalline KNO_3_ occurring around 334 °C [21].

Generally, compounds with higher ionic mobility should have lower fusion temperatures due to the tendency of ions to overcome the forces holding the lattice together. However, KNO_3_ has a relatively high melting point (334 °C) because of strong electrostatic forces between the K^+^ and NO_3_^−^ ions, which create a stable lattice structure in the solid state. However, if the ionic mobility were significantly higher, the lattice could be easily disrupted, leading to a lower melting point.

### 3.3. TGA

**Figure 5:**
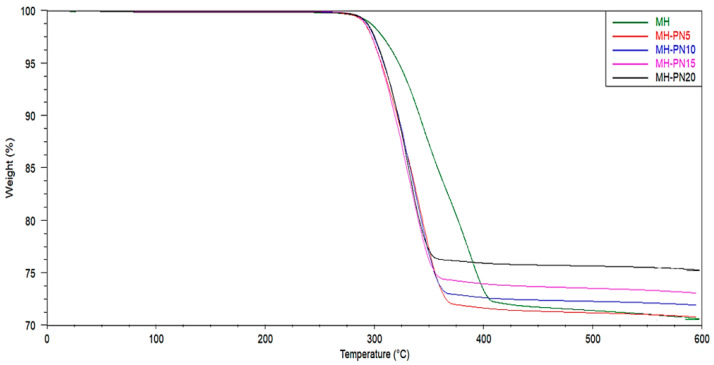
TGA overlay curves for the pure and doped Mg(OH)_2_.

**Figure 6:**
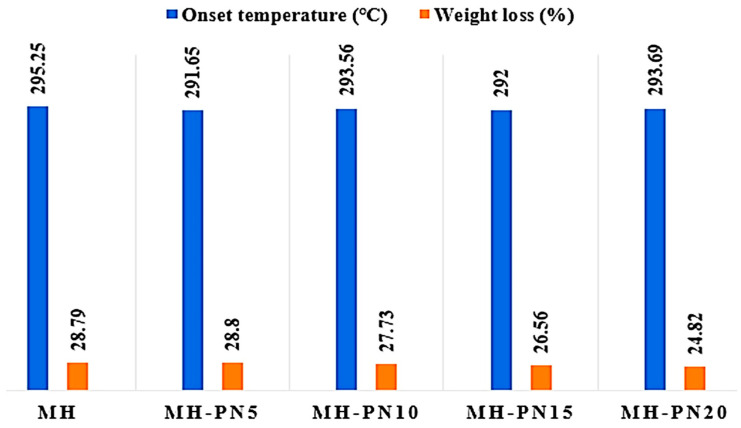
Thermogravimetric onset temperatures and weight loss in the pure and doped Mg(OH)_2_.

TGA was carried out to provide an overview of the thermal stability of the materials during dehydration by observing the weight variation with temperature ramping at a constant rate. The TGA overlay curves for pure and doped Mg(OH)_2_ samples are shown in Figure 5, while the summary of the results is presented in Figure 6. For pure Mg(OH)_2_, the weight loss at 295.25 °C is 28.79%, and for the composites, their thermal stabilities are closely related to that of pure material. For instance, the average difference in weight loss is around 4%, whilst the average difference in onset temperature is ~3 °C. These marginal differences suggest that the thermal integrity of the pure material is preserved even with the addition of KNO_3_. Specifically, the MH-PN5 composite has a weight loss of 28.80% compared to 28.79% for pure material, MH.

The overall analysis of the DSC/TGA results showed that sample MH-PN5, which represents Mg(OH)_2_ with 5 wt% KNO_3_ addition, is the only composite that could reduce the dehydration temperature as well as increase the energy storage capacity of the pure Mg(OH)_2_, whilst maintaining good thermal integrity. Therefore, further physical characterizations of the MH-PN5 composite were carried out.

### 3.4. BET Analysis of MH-PN5

**Figure 7:**
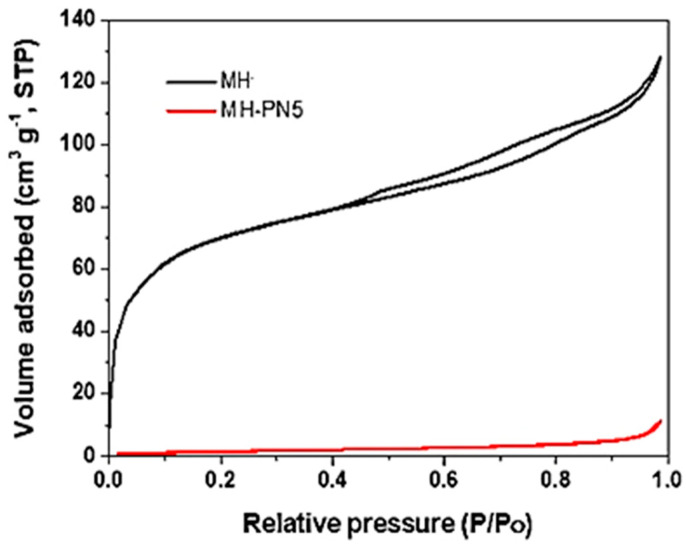
Adsorption isotherms for pure MH and MH-PN5 composite.

The BET surface areas (S_BET_) of the pure (MH) and 5 wt% KNO_3_-doped (MH-PN5) samples are presented in Table 1. It shows the pure Mg(OH)_2_ with a high S_BET_ value of 250 m^2^/g and the MH-PN5 composite with a value of 6 m^2^/g. The BET result for the doped sample (MH-PN5) shows a dramatic reduction in its surface area compared with the pure MH sample. This could be attributed to the pore-plugging effect of the dopant, leading to a decline in porosity, but not sufficient for agglomeration. In Figure 7, the nitrogen adsorption profile for pure MH appears as a type IV isotherm, indicating some level of mesoporosity. However, the doped sample did appear as type III isotherms.

### 3.5. PSD Analysis of MH-PN5

**Figure 8:**
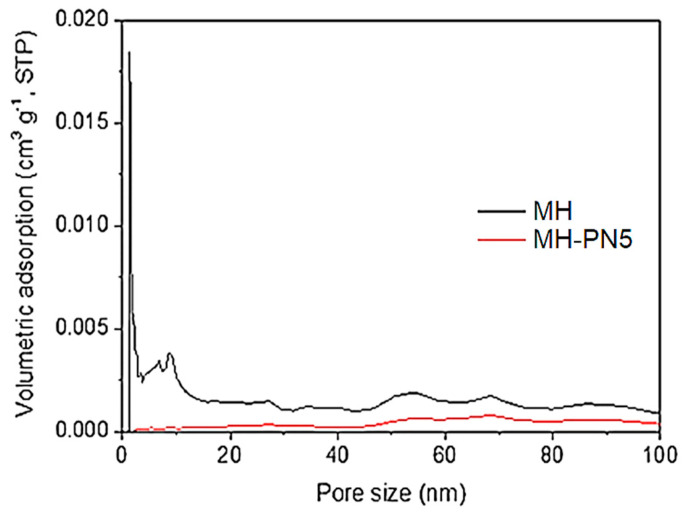
PSD for pure MH and MH-PN5 composite.

The wide disparity in the S_BET_ values of the samples was further investigated by comparing their pore size distribution (PSD) with and without doping. The PSD curves for the pure MH and the doped (MH-PN5) materials are shown in Figure 8. The curves show that MH has some microporosity, with pore sizes less than 2 nm, and large mesoporosity, with pore sizes between 2 and 40 nm. Thus, the dramatic reduction in the surface area is due to the dopant’s pore-clogging effect, especially at the micropores, as they become inaccessible to the probe molecule (N_2_) during adsorption. As can be seen in Figure 8, pores smaller than 20 nm are present in the MH but significantly reduced after doping. This could impede the sorption capacity of this material since sorption depends on the degree of porosity, surface area, and pore volume [22].

### 3.6. SEM Analysis of MH-PN5

**Figure 9:**
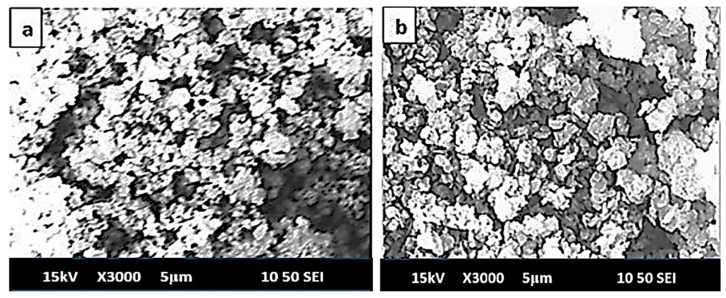
SEM images for (**a**) pure MH and (**b**) MH-PN5 composite.

The SEM image of the pure Mg(OH)_2_ (MH) is shown in Figure 9a. This material had a fluffy or spongy morphology with closely arranged particles. This spongy texture is suggestive of a large surface area, as adequately supported by the S_BET_ value of 250 m^2^/g. This would have the advantage of adequate heat adsorption capacity. The addition of 5 wt% KNO_3_ (MH-PN5) results in monodisperse quartzite-shaped grains (Figure 9b). The monodispersity is an indication that the constituent atoms are uniformly distributed in the material with minimal variation in size and shape. However, the micropore spaces are close, which explains the low S_BET_ value that could negatively impact the heat adsorption capacity of the composite material.

### 3.7. EDX Analysis of MH-PN5

**Figure 10:**
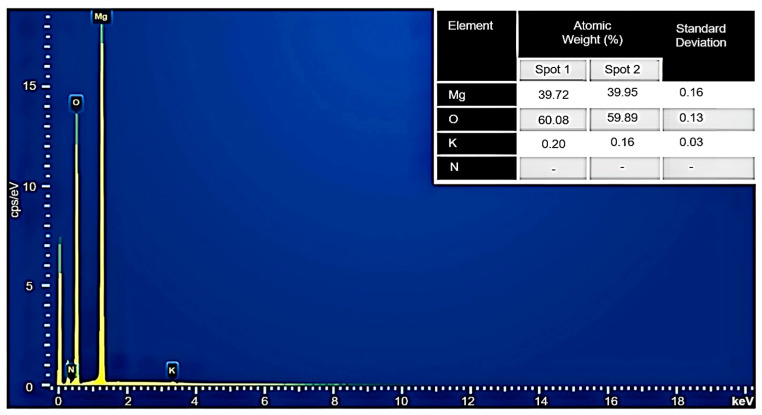
EDX spectra and data for the MH-PN5 composite.

Figure 10 shows the EDX spectra for the MH-PN5 composite, consisting of quantitative data at each sampled point, spots 1 and 2. The EDX of the elemental compositions of Mg, O, and K have values of 39.72, 60.08, and 0.20% at sampled spot 1 and 39.95, 59.89, and 0.16% at spot 2, showing consistency (approximately the same values) in the sampled areas. These correspond to atomic weights of 49.92, 49.68, and 0.40% at spot 1 and 50.17, 49.50, and 0.33% at spot 2 for Mg, O, and K, respectively. The nitrogen (N) element was not detected, as is sometimes the case with small quantities of light atoms. The very small standard deviation values of 0.16, 0.13, and 0.03 for Mg, O, and K, respectively, are indicative of the uniform atomic weight distribution in the material. Thus, the EDX supports the monodisperse SEM topographic information about this composite material.

## 4. Conclusions

This work was aimed at doping Mg(OH)_2_ with KNO_3_ for medium-temperature heat storage application. The XRD results showed clear traces of the core materials and the doping agent, thus confirming the success of the synthesis. The DSC/TGA results revealed that 5 wt% KNO_3_ had a more positive impact on the Mg(OH)_2_ in terms of reduction in dehydration temperature, heat storage enhancement, and thermal stability, and was therefore found to possess the highest potential for heat storage within the medium-temperature range of 293–400 °C. However, the BET test conducted on the MH-PN5 sample showed a reduction in the surface area and porosity, which was attributed to the dopant’s pore-plugging effect. Analysis of the SEM results also showed that the micropore spaces were close and could therefore have a negative impact on the heat adsorption capacity of the composite material, but not enough to agglomerate. The EDX results also supported the monodisperse topographic structure shown in the SEM. Despite the negative physical adsorption characteristics, this study has established sample MH-PN5 as a promising candidate for medium-temperature heat storage. Hence, there is a need to pursue further enhancements of this material aimed at attaining prolonged storage capacity and cyclability. It would be valuable if, in the future, the most suitable material was chosen for an in-depth study of its dehydration kinetics and studied in conditions typical for TCES cycles. This will be a more realistic way to determine its suitability for practical application.

## Figures and Tables

**Table 1:** BET surface areas of the samples.

Sample	S_BET_ (m^2^/g)
MH	250
MH-PN5	6

## Data Availability

All the data are available in this manuscript.
